# Successful use of human amniotic allograft membrane in the treatment of recalcitrant driveline infections

**DOI:** 10.1016/j.jhlto.2025.100351

**Published:** 2025-07-25

**Authors:** Cristiano Spadaccio, Udit Vyas, Antonio Panza, Russell Samuel Vester, Louis Benson Louis IV

**Affiliations:** a Department of Cardiac Surgery, University of Cincinnati College of Medicine, Cincinnati, OH; b Department of General Surgery, Wright State University Boonshoft School of Medicine, 30 E Apple St. Dayton, Ohio 45409

**Keywords:** Driveline infection, Left ventricular assist device (LVAD), Human amniotic allograft membrane

## Abstract

Driveline infections represent a significant source of morbidity and mortality in left ventricular assist device (LVAD) patients. Driveline infection occur in up to 40% of cases and have recurrence rates up to 50%. Current literature fails to demonstrate specific interventions capable of reducing prevalence of recalcitrant driveline infections (including serial debridement, muscle flap coverage of the driveline, driveline relocation, and device exchange). Human cryopreserved decellularized amniotic membrane (HCDAM) has been effectively used in chronic non-healing infected diabetic ulcers and reconstructive orthopedic and ophthalmology surgery for its advantageous biological properties. With no univocal consensus treatment option, we firstly explored HCDAM application in recalcitrant driveline infection and we describe the successful outcome in a patient who failed previous management with serial debridement. By wrapping HCDAM around the driveline we were able to obtain a positive tissue response leading to infection resolution.

Left ventricular assist devices (LVADs) are an increasingly prevalent and critical tool in the management of cardiac disease as circulatory support for myocardial recovery and as a treatment tool in end-stage heart failure.^1^ Despite the significant technological advancement and improvement in clinical outcomes, driveline infections remain a prevalent and cumbersome complication; occurring in up to 40% of cases and accounting for major morbidity, readmissions, and reduced long-term survival.^1^ The driveline serves as a nidus for infection, as biofilm formation leads to colonization by highly resistant organisms (in particular Staphylococcus and Pseudomonas species) with progression to bacteriemia and life-threatening sepsis.^2^, ^3^

Interventions to prevent or mitigate the risk of driveline infection have been attempted with inconsistent results and large variability in practice across the centers.^4^ When colonization occurs, recalcitrant infections represent a significant challenge with a variety of potential solutions: serial wound debridements, use of a muscle flap for coverage of the driveline, relocation of the driveline, and even device exchange. Nevertheless, the recurrence rate of infections can be as high as 50%.^5^

In recent years, human cryopreserved amniotic membrane and umbilical cord have been proposed as a means of improved wound healing in light of their immunomodulatory properties with encouraging results.^6^, ^7^ Amniotic membrane material retains the ability to produce cellular signaling factors thus reducing inflammation and promoting a more harmonic tissue healing and regeneration.^8^ The cellular signaling factors improve wound healing through a variety of proposed mechanisms including: stimulation of angiogenesis, secretion of antimicrobial peptides (i.e beta-defensins), production of cytokines which inhibit inflammation and thus reduce fibrosis, and production of cytokines which have immunomodulatory effects and prevent cellular apoptosis.^7^, ^8^, ^9^, ^10^ Further, amniotic membrane lacks expression of many HLA antigens and consequently has low immunogenic property.^10^ Additionally, amniotic tissues have been shown to have antimicrobial properties given their ability to express antimicrobial molecules such as human beta-defensins and elafsin.^9^ Further, the ECM provides an antimicrobial shield against antimicrobial infiltration as well as expression of antimicrobial peptides which function by compromising microbial membranes and suppressing pathogenic proliferation.^11^, ^12^, ^13^, ^14^ Through these mechanisms, it has been proposed that human cryopreserved amniotic membrane encourages native tissue regeneration thus facilitating accelerated wound healing; and this theory has been successfully demonstrated in applications such as spina bifida repairs, non-healing diabetic ulcers, and in ankle arhtroplasties.^7^, ^8^, ^9^, ^10^

We therefore explored the hypothesis of a potential use of cryopreserved amniotic membrane in recalcitrant driveline infection and we here report the first in-human use with successful outcome.

The clinical application occurred in a 33-year-old male with morbid obesity (BMI 52 kg/m^2^) who underwent placement of LVAD (HeartMate 3; Abbott; Abbott Park, IL) in January 2023 for end-stage non-ischemic cardiomyopathy as destination therapy. Over the course of his therapy bridging to heart transplant he was engaged in a multi-disciplinary team that included cardiology, advanced nutrition specialists, and a referral to bariatrics with a total weight loss of 15% of body mass. His post-op course was complicated by multiple recalcitrant driveline infection requiring 6 operative debridement procedures. Multiple organisms were found repeatedly to be causative including Actinobacter Hemolyticus, Pseudomonas Aeruginosa, and Staphylococcus Epidermis at each debridement procedure. His antimicrobial regimen was progressively tailored and included multiple antibiotics administered both intravenously and orally over the course of these months. These included originally Levofloxacin and Ampicillin/Sulbactam, then Cefepime and Vancomycin, followed by Doxycycline and Meropenem which was then transitioned to ceftolozane and tazobactam because of meropenem-resistant Pseudomonas Aeruginosa at tissue cultures. In early October 2024, he presented with recurrent driveline infection with purulent discharge from the driveline insertion site, and abscess formation within the subcutaneous tissue and fascia with loss of substance, but no bacteriemia. Because of the repeated failure of standard surgical debridement and vacuum-assisted closure systems (VAC), we explored the option of using a human amniotic membrane allograft to assist with wound healing. At the time of surgery blood cultures were negative and CT scan revealed concern for contiguous spread of infection into abdominal cavity. After informed consent was obtained, an excisional debridement of the infected driveline exit site was performed. A circumferential incision was carried down to the fascia of the anterior rectus sheath. Infected rectus abdominis muscle and part of the posterior rectus sheath were removed leaving a wound of approximately 4×4 cm in size and 4 cm in depth(Figure 1A). After extensive debridement and copious irrigation with gentamicin saline solution, a 6×3 cm ultra-thick amniotic membrane allograft (Clarix 1 K; BioTissue, Miami, FL, USA) was used to wrap the driveline at the base and placed this around the driveline below the rectus sheath(Figure 1B). The velour of the driveline was embedded in dense inflammatory scar tissue, and despite multiple attempts, was not able to be excised without causing driveline damage; this was likely due to the multiple previous debridements that had been performed as part of his course. The procedure was completed with the standard application of a wound-VAC with cultures from removed tissues growing multidrug resistant Pseudomonas Aeruginosa. At the first VAC change 48 h after the procedure, formation granulation tissue in the wound bed around the implanted amniotic membrane could be observed (Figure 2).

**Figure 1:**
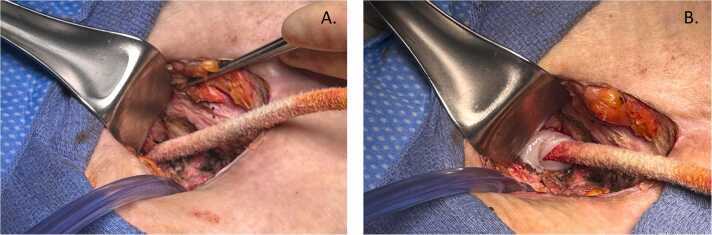
A: intraoperative image depicting the wound after initial operative debridement. Approximate dimensions 4×4cm and 4 cm in depth at the level of the posterior rectus sheath; B: application of amniotic allograft around the driveline.

**Figure 2:**
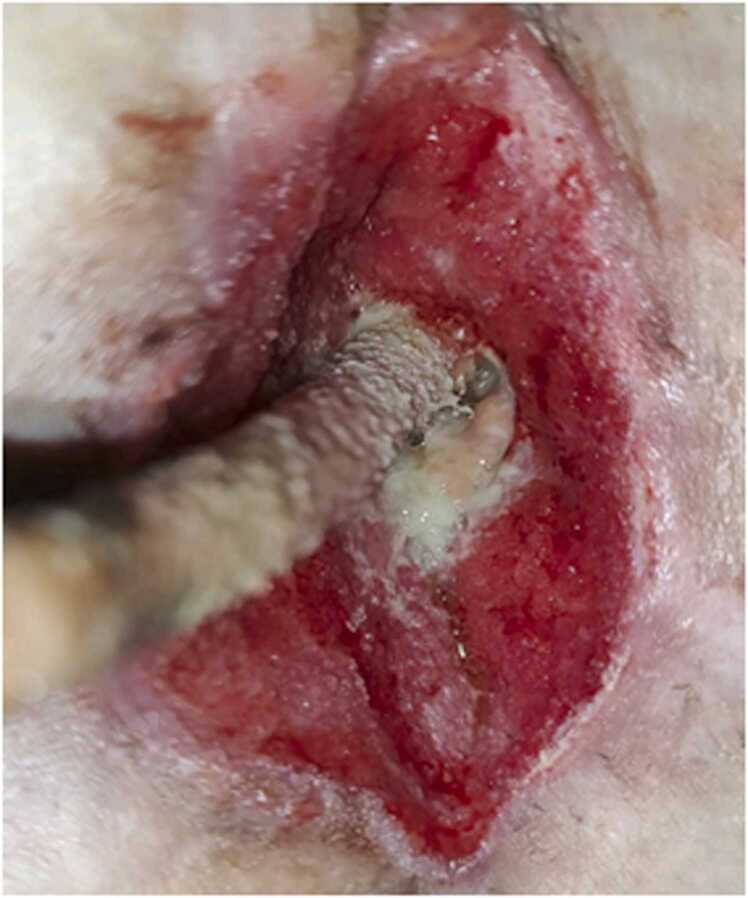
Wound at the first VAC change 48 h after the procedure. Notably, there is already formation of healthy granulation tissue around the allograft material.

Over the next 6 weeks, the patient underwent planned serial VAC exchanges and Figure 3 demonstrates wound progression over this timeframe, with progressive resorption of the membrane and accompanying tissue response until closure of the dermal layers (Figure 3F**,** approx. 40 days after allograft application). During this time, patient was able to be discharged home without infection recurrence and continuing dermal healing. The patient was able to be listed for transplant at another institution at the end November 2024, 45 days after the amniotic allograft implant. Transplant and LVAD explant occurred a week later and at the time of surgery, no evidence of infection was found intraoperatively on the mediastinum, driveline trajectory and driveline itself, and therefore no tissue cultures were obtained. Following the transplant, the wound reached complete closure including the epidermal layers, approximatively 60 days since the original application of the amniotic allograft (Figure 4).

**Figure 3:**
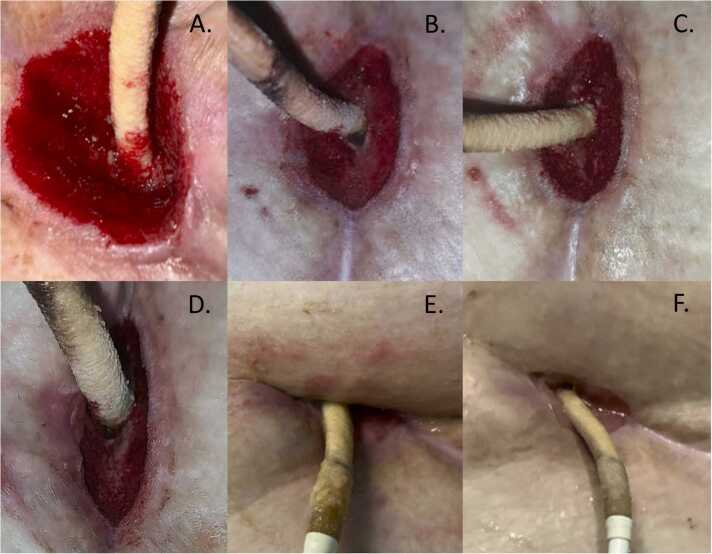
Serial photographic documentation showing the progression of wound healing during 6-week post-operative course with formation of granulation tissue around allograft and progressive closure of wound.

**Figure 4:**
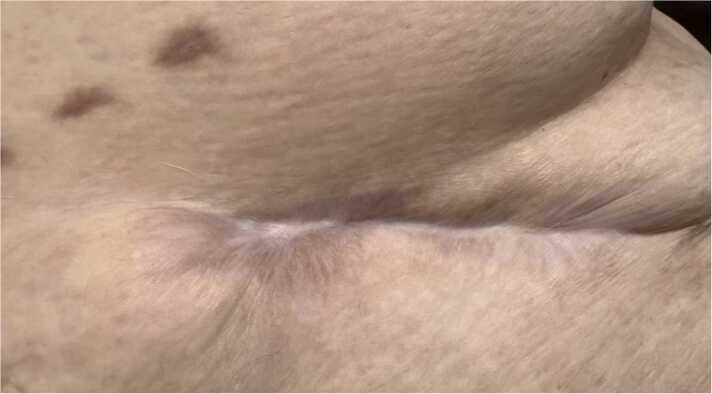
Final wound closure approximately 60 days after application of amniotic allograft material and after transplantation with LVAD and driveline explant.

Recalcitrant driveline infections continue to plague the outcomes of VAD implantation^1^ and a large survey-based study has largely demonstrated the lack of effectiveness of the majority of the preventative strategies previously utilized.^4^ Also, advanced antibiotic delivery approaches, as antibiotic beads, have been shown to be partially successful, requiring multiple bead exchange procedures and months of therapy to achieve eradication.^9^

Amniotic and umbilical cord materials have recently emerged as a valid adjunct in tissue regeneration as promoting an anti-inflammatory environment conducive to a harmonic and expedited tissue repair process. These materials have been used in a variety of fields including non-healing diabetic ulcers, orthopedics, and neurosurgery demonstrating their ability to promote native tissue regeneration, stimulate granulation tissue formation, prevent fibrosis and provide expedited wound healing.^7^, ^10^ The amniotic membrane exhibits intrinsic antimicrobial activity through the expression of antimicrobial peptides (AMPs) which disrupt microbial membranes and inhibit pathogen proliferation. Beyond its barrier function it modultaes immune celle activity, fostering a pro-healing micronenvironment that support tissue regeneration.^9^, ^11^, ^12^, ^13^, ^14^

We here firstly propose and demonstrate the successful clinical use of human amniotic membrane to promote healing of recalcitrant driveline infection. The use of this material in association with vacuum-assisted wound management system resulted in the resolution of a highly resistant and recurring infection of a HM-3 driveline unresponsive to a variety of treatments and repeatedly failing to heal despite multiple standard surgical debridement plus VAC procedures. The combination of VAC and amniotic membrane application allowed wound healing within 6 weeks with no further infection recurrence. As observed in previous non-cardiac uses of this material, the amniotic membrane appeared to aid in granulation and stimulate the formation of healthy, viable tissue with a single operative intervention and no further infection recurrence.

Considering the nature of this report and the lack of a control arm utilizing standard surgical debridement and VAC therapy-only, it is not possible to discern the relative role of the amniotic tissue in the healing process and to distinguish whether its presence expedited timing of wound healing. However, considering the previous repeated failure of standard surgical debridements with VAC closure and the multiple reports of effectiveness in other medical disciplines, we can reliably speculate that the application of the amniotic membrane may have contributed to the positive outcome. Furthermore, unlike with antibiotic bead exchanges, this case required a single intervention.

Despite the anecdotal nature of this report, this successful outcome paves the way for a potential use of amniotic membranes in driveline-related infections. The authors acknowledge that this case represents a single report, and that the timeframe of the case (6 weeks until transplant) makes determining the long-term effectiveness of amniotic membranes difficult. However, in this report we present the first human case of using human amniotic membrane as a means of accelerating resolution of local infection. The findings of this report are encouraging (especially given history of multi-drug resistant organism infections) but preliminary; the case demonstrates a promising but speculative outcome. In this report we propose a novel methodology for further investigation. Whether this approach could be also considered as preventative measures at the time of the first VAD implant remains uncertain but plausible and would warrant larger case-control investigations.

## Consent

Informed consent was obtained from the patient prior to implanting the novel allograft material; the informed consent process included a full risks, benefits, and alternatives discussion and the patient consented to implantation as well as inclusion in case reports and publications if successful.

## Declaration of Competing Interest

The authors declare that they have no known competing financial interests or personal relationships that could have appeared to influence the work reported in this paper.
